# Prognostic significance of E-cadherin and ZEB1 expression in intraductal papillary mucinous neoplasm

**DOI:** 10.18632/oncotarget.23012

**Published:** 2017-12-07

**Authors:** Ye Rim Chang, Taesung Park, Sung Hyo Park, Yong Kang Kim, Kyoung Bun Lee, Sun-Whe Kim, Jin-Young Jang

**Affiliations:** ^1^ Department of Surgery and Cancer Research Institute, Seoul National University College of Medicine, Seoul, Korea; ^2^ Department of Surgery, Dankook University College of Medicine, Cheonan, Korea; ^3^ Department of Statistics, Seoul National University College of Natural Sciences, Seoul, Korea; ^4^ Department of Pathology, Seoul National University College of Medicine, Seoul, Korea

**Keywords:** epithelial-to-mesenchymal transition, intraductal papillary mucinous neoplasm, ZEB1, differentially expressed gene, biomarker

## Abstract

There is an urgent need to investigate the genetic changes that occur in intraductal papillary mucinous neoplasm (IPMN), which is a well-known precursor of pancreatic cancer. In this study, gene expression profiling was performed by removing unwanted variation to determine the differentially expressed genes (DEGs) associated with malignant progression of IPMN. Among the identified DEGs, zinc finger E-box binding homeobox 1 (ZEB1) and E-cadherin, a crucial regulator of epithelial-to-mesenchymal transition (EMT), was validated among identified DEGs.

A total of 76 fresh-frozen tissues were used for gene expression profiling and formalin-fixed, paraffin-embedded blocks from 87 patients were obtained for immunohistochemical analysis. Loss of E-cadherin expression (*p* = 0.023, odd ratio [OR] = 4.923) and expression of ZEB1 in stromal cells (stromal ZEB1, *p* < 0.001, OR = 26.800) were significantly correlated with degree of dysplasia. The hazard of death was significantly increased in patients with loss of E-cadherin expression (hazard ratio [HR] = 13.718, *p* = 0.004), expression of epithelial ZEB1 (HR = 19.117, *p* = 0.001), and stromal ZEB1 (HR = 6.373, *p* = 0.043).

Based on the results of this study, loss of E-cadherin and expression of stromal ZEB1 are associated with increased risk of malignant progression. Epithelial and stromal ZEB1, as well as E-cadherin may be strong predictors of survival in patients with IPMN. Our finding suggests that these EMT markers may be utilized as potential prognosticators and may be used to improve and personalize treatment of IPMN.

## INTRODUCTION

Intraductal papillary mucinous neoplasm (IPMN) has been increasingly recognized as an important cystic precursor of pancreatic cancer [1]. It has been suggested that IPMN undergoes a progression pattern, which consists of hyperplasia, dysplasia, and invasive carcinoma sequence [2, 3]. Numerous efforts have been made to identify the genetic changes associated with progression of IPMN. Since preoperative assessment of gene expression profiling has been used to differentiate invasive from noninvasive IPMNs [4], *GNAS* and *KRAS* mutations have been identified by direct sequencing [5], and immunohistochemical analysis suggested correlations between fascin overexpression and increased histological grade of IPMN [6]. Copy number gain of chromosome 3q was also found to be associated with IPMN progression [7]. Loss of expression of PTEN [8] and Plectin-1 [9] have also been reported to be associated with poor prognosis or malignant progression of IPMN.

However, despite the previous efforts, the molecular mechanism involved in the malignant progression in IPMN remains unknown. Moreover, many lesions are heterogeneous, with varying degrees of dysplastic and invasive regions within the same specimen [10]. Adequate sampling is crucial for recognition of invasive carcinoma, as it may be present in only as small part of the lesion. Current consensus guidelines still rely mostly on radiologic findings to detect signs of high-grade or invasive lesions, which commonly lead to misdiagnosis of its invasiveness. The role of imaging modalities to predict cancer progression and individualize patient management is limited, since they do not predict risk of malignant transformation [11]. Therefore, there is an urgent need to investigate the genetic changes of carcinogenesis of IPMN in order to improve the diagnosis and management of IPMN. In this study, gene expression profiling was performed to determine differentially expressed genes (DEGs) associated with malignant progression of IPMN; the prognostic significance of the identified DEGs, as well as their potential as biomarkers, were evaluated.

## RESULTS

### Patient characteristics

For gene expression profiling, 76 IPMN samples consisted of 47 gastric, 16 intestinal, 11 pancreatobiliary, and 2 oncocytic subtypes were used. Samples included low-grade (*n =* 11), intermediate-grade (*n =* 31), high-grade dysplasia (*n =* 14), as well as IPMN with an associated invasive carcinoma (invasive IPMN, *n =* 20). Patients consisted of 46 men (63.9%) with a median age of 65.5 ± 7.7 years at the time of diagnosis. For final analysis, low-grade dysplasia (*n =* 11) and invasive IPMN samples (*n =* 9) of the gastric subtype were compared; this included 11 men (64.7%) with a median age of 63.0 ± 6.5 years at the time of diagnosis. The diameter of the tumor ranged between 2.4-7.6 cm (median 3.1 cm); and the number of main duct, branch duct, and mixed type cases were 2, 10, and 5, respectively. Lymph node metastasis was identified in 1 out of 9 invasive IPMNs.

Detailed clinicopathological data of patients whose samples were subjected to immunohistochemical analysis are presented in Table 1. Of the 87 patients, pancreas head resection was performed in 44 cases, distal resection and limited pancreatic resection were conducted in 27 and 7 cases, respectively. Curative resection was performed in 86 cases except for one patient, who showed peritoneal seeding. Invasive carcinoma was diagnosed in 20.7% of the cases, and lymph node metastasis was found in 4 patients. The histologic subtype of the study patients were as follows: 52 (59.8%) gastric types, 17 (19.5%) pancreatobiliary types, 16 (18.4%) intestinal types, and 2 (2.3%) oncocytic types. Overall 5 year disease-specific survival rate of patients was 72.2%. No disease-related death was observed in low, moderate, and high grade dysplasia IPMN. Among the 18 patients with invasive IPMN, 5 patients died as a result of IPMNs, and 2 patients died owing to other types of cancer. During the 51.8 months of median follow-up period, recurrence was observed in 9 patients: 1 patient with low-grade dysplasia, 2 patients with intermediate-grade dysplasia, and 6 patients with invasive IPMN.

**Table 1:** Clinicopathological characteristics of 87 IPMNs included in immunohistochemical analysis

Parameter	*n* = 87
Age (years, median ± SD)	65.0 ± 8.3
Sex (male)	53 (60.9%)
Dysplasia	
Low grade	15 (17.2%)
Intermediate grade	40 (46.0%)
High grade	14 (16.1%)
IPMN associated with an invasive carcinoma	18 (20.7%)
Histologic subtype	
Gastric	52 (59.8%)
Pancreatobiliary	17 (19.5%)
Intestinal	16 (18.4%)
Oncocytic	2 (2.3%)
Operative methods	
Whipple’s operation/PPPD	13/31 (50.6%)
DP/SPDP	24/3 (31.0%)
Total/subtotal pancreatectomy	5/4 (10.3%)
Enucleation	4 (4.6%)
Others^*^	3 (3.4%)
Cyst size (mm, median ± SD)	28.0 ± 17.0
≥30 mm	40 (46.0%)
Diameter of main pancreatic duct (mm, median ± SD)	3.3 ± 5.2
<5 mm	52 (59.8%)
≥5, <10 mm	21 (24.1%)
≥10 mm	14 (16.1%)
T stage	
T is/1/2	14/1/5 (23.0%)
T3/4	11/1 (13.8%)
Lymph node metastasis	4 (4.6%)
Distant metastasis	1 (1.1%)
5 year disease-specific survival	94.3%
Noninvasive (*n* = 69)	100.0%
IPMN associated with an invasive carcinoma (*n* = 18)	72.2%

^*^Pancreatic head resection with segmental duodenectomy (*n* = 2), Duodenal-preserving resection of the head of the pancreas (*n* = 1)

SD, standard deviation; IPMN, intraductal papillary mucinous neoplasm; PPPD, pylorus-preserving pancreaticoduodenectomy; DP, distal pancreatectomy; SPDP, spleen-preserving distal pancreatectomy

### Differentially expressed gene analysis

**Figure 1:**
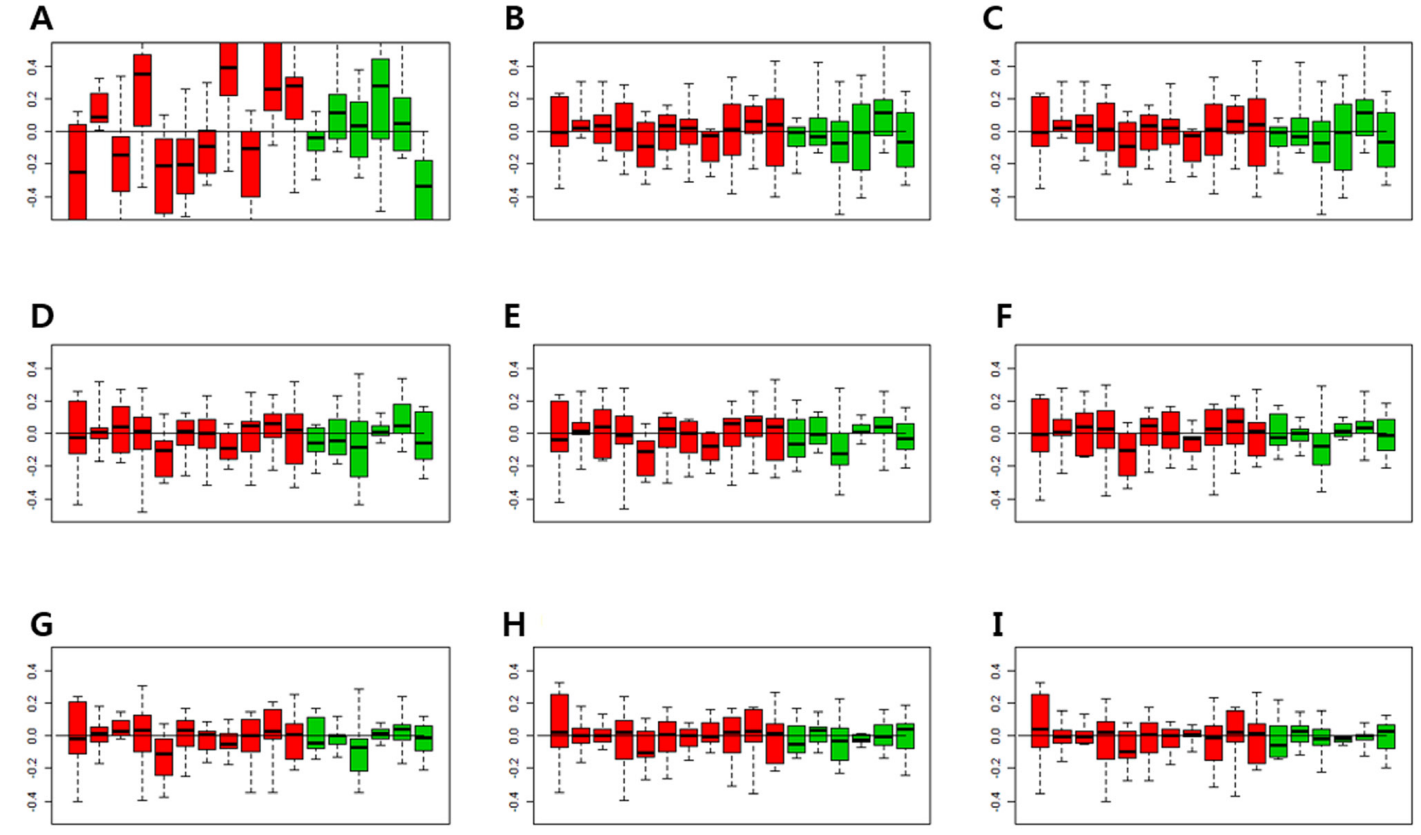
Relative log expression (RLE) plots for all control genes The RLE plots of control genes with 1-8 factors were compared to determine the appropriate number of factors that were consistent with the gene expression patterns of control genes. (**A**) RLE plot by the RMA normalization. (**B**) RLE plot normalized by RUV, number of factors (κ) = 1. (**C**) RLE plot normalized by RUV, κ = 2. (**D**) RLE plot normalized by RUV, κ = 3. (**E**) RLE plot normalized by RUV, κ = 4. (**F**) RLE plot normalized by RUV, κ = 5. (**G**) RLE plot normalized by RUV, κ = 6. (**H**) RLE plot normalized by RUV, κ = 7. (**I**) RLE plot normalized by RUV, κ = 8. (Red boxplots indicate low-grade dysplasia and green boxplots indicate invasive IPMNs. Lines inside the boxplots indicate median values).

In order to find the minimal number of factor that can consistently match the gene expression pattern of the control genes, the relative log expression (RLE) boxplots of the control genes were obtained (Figure 1). The expressions of the control genes were not properly normalized using the Robust Multi-Array Average (RMA) normalization (Figure 1A). When Removing Unwanted Variation (RUV) was performed, the median of RLE boxplots of all samples became almost constant (Figure 1B–1I). When the number of factors was more than 3, the inter quartile range (IQR) values ​​of the control gene became similar (Figure 1D–1I). Since one cannot completely match the number of the factors with gene expression patterns, and the expression patterns of the control genes within IQR levels can be adjusted by RUV, the optimal number of factors was considered to be 3–5.

**Figure 2:**
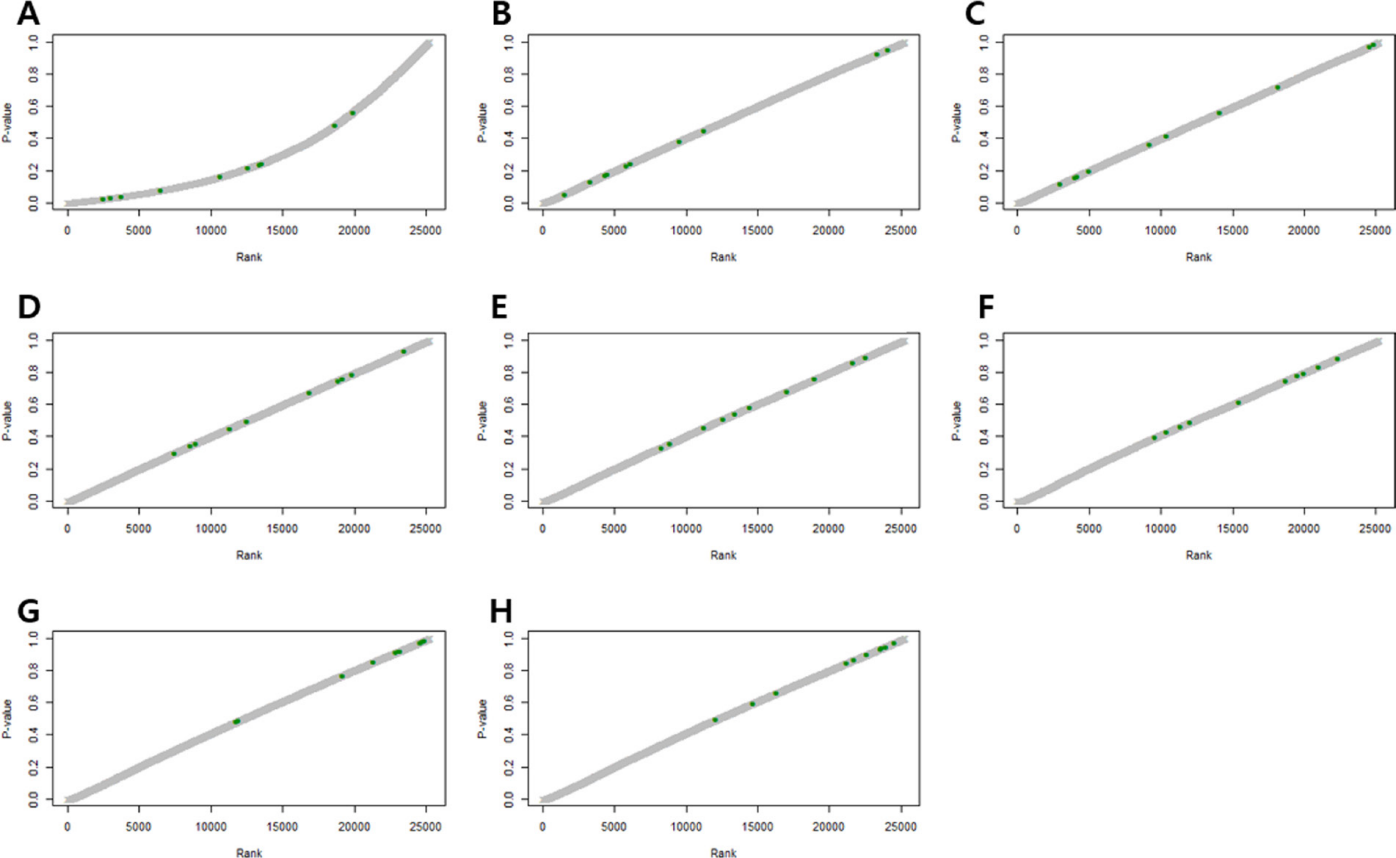
*P*-value plots of control genes (**A**) Unadjusted *P*-value plots. Some *p*-values of the control genes are less than 0.05. (**B**) *P*-value plots normalized by RUV, κ = 1. (**C**) *P*-value plots normalized by RUV, κ = 2. (**D**) *P*-value plots normalized by RUV, κ = 3. (**E**) *P*-value plots normalized by RUV, κ = 4. (**F**) *P*-value plots normalized by RUV, κ = 5. (**G**) *P*-value plots normalized by RUV, κ = 6. (**H**) *P*-value plots normalized by RUV, κ = 7. (Green dots indicate control genes).

**Figure 3:**
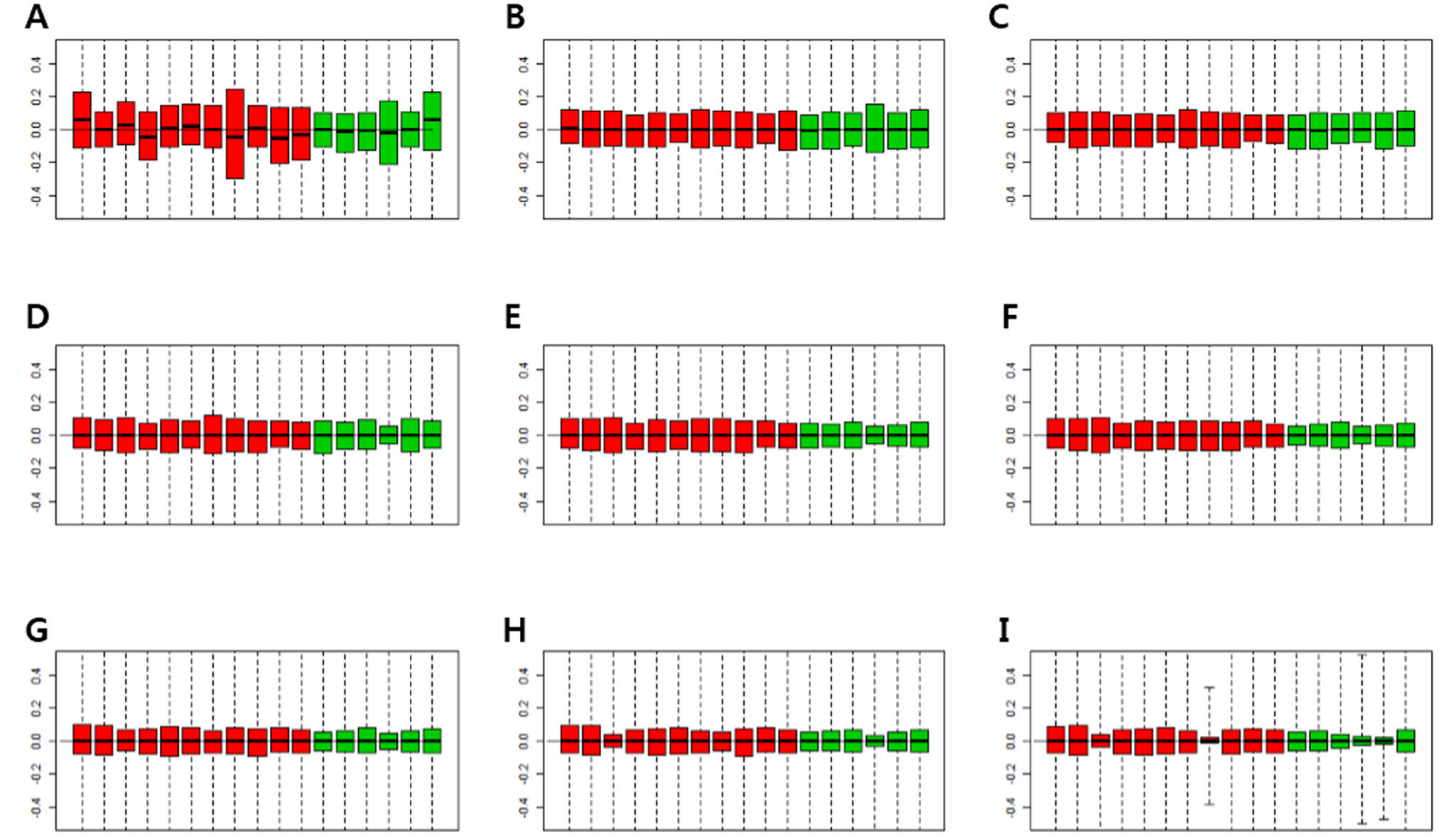
Relative log expression (RLE) plots for all genes (**A**) RLE plot by RMA normalization. (**B**) RLE plot normalized by RUV-4 method with number of factors (κ) = 1. (**C**) RLE plot normalized by RUV-4, κ = 2. (**D**) RLE plot normalized by RUV-4, κ = 3. (**E**) RLE plot normalized by RUV-4, κ = 4. (**F**) RLE plot normalized by RUV-4, κ = 5. (**G**) RLE plot normalized by RUV-4, κ = 6. (**H**) RLE plot normalized by RUV-4, κ = 7. (**I**) RLE plot normalized by RUV-4, κ = 8. (Red boxplots indicate low-grade dysplasia, green boxplots indicate invasive IPMN. Lines inside the boxplot indicate median values).

Figure 2 shows *p*-values of DEGs obtained by the RUV-4 method, with an increase in the number of factors. *P*-values of some control genes were less than 0.05 before RUV was performed (Figure 2A). After RUV-4 was performed, the control genes with *p*-value of less or equal to 0.2 were identified when the number of factors was 1 or 2 (Figure 2B, 2C). The *p*-values of control genes were greater or equal to 0.2 when more than 4 factors were used (Figure 2E–2H). When the number of factors was greater than 6, the control genes lost too much variation, and the *p*-value tended to become extremely high (Figure 2G, 2H). Therefore, Figure 2D seemed appropriate. Figure 3 shows the RLE plot of all genes. When the number of factors was greater than 7, the range of gene expression in some samples became much smaller as compared with that in other samples (Figure 3H, 3I). These results suggested that the optimal number of factors required to maintain the expression patterns of DEGs while adjusting for expression patterns of the control gene was 3–5 (Figure 3A–3G). Since the RUV method cannot eliminate all unwanted variations, and fitting too many factors causes biases, it was decided to use 3 factors to perform RUV-4.

**Figure 4:**
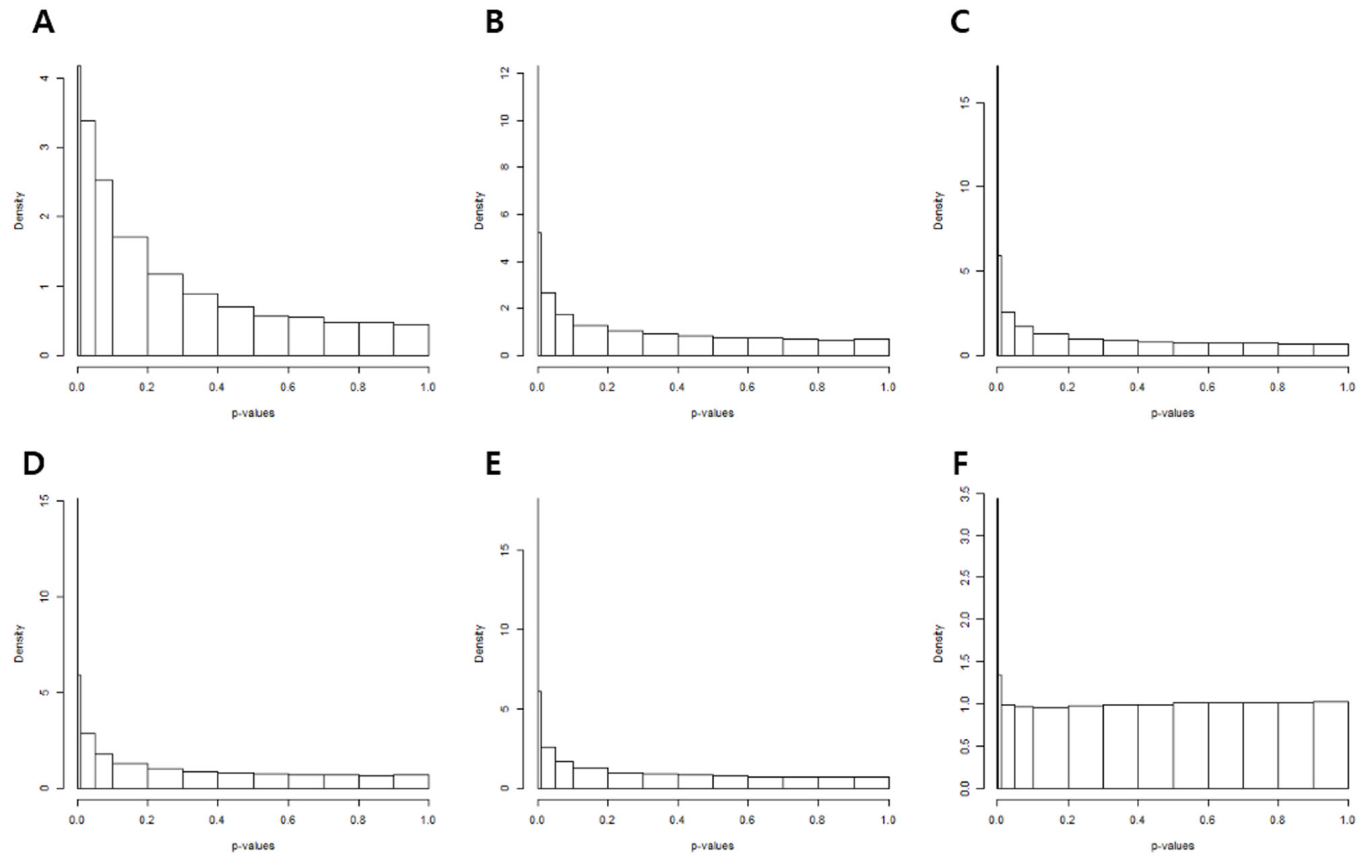
*P*-value histograms of each removing unwanted variation methodology (**A**) The histogram obtained from *t*-tests show exponential distribution. The variance of the statistic was calculated by (**B**) the standard method, (**C**) empirical bayes, (**D**) rescaled variance, and (**E**) rescaled variance with empirical Bayes. (**F**) Distribution and peak of *p*-values when variance of the statistic was calculated by the empirical variance

The *p*-value histograms of each methodology used to calculate the *p*-value is shown in Figure 4. The histogram obtained from *t*-tests exhibited an exponential distribution (Figure 4A). This suggested that unwanted variations of the control genes caused *p*-value deflation when *t*-tests were performed. Variance calculation using statistical methods such as the standard, empirical Bayes (e-bayes), rescaled variance (rsvar), and rescaled variance with empirical Bayes (rsvar e-bayes) demonstrated uniform distribution when *p*-value was greater or equal to 0.1 (Figure 4B–4E). However, extremely low *p-*values were obtained for many genes, which implied that there were more false positives present owing to calculation error of the variance of the statistic. When the variance of the statistic was calculated using the empirical variance, the distribution of the *p*-value (≥ 0.01) was uniformly adjusted. In addition, the peak was found only in *p*-values that were less or equal to 0.01 (Figure 4F). Therefore, DEGs were established by the RUV-4 method with 3 factors and empirical variance adjustments. Table 2 lists the top 10 DEGs, of which the *q*-values were less than 0.05. Among the listed DEGs, zinc finger E-box binding homeobox 1 (ZEB1), which is a crucial regulator of EMT, was selected for validation of results. E-cadherin and vimentin were also validated to figure out an association of EMT with the prognosis of IPMN.

**Table 2:** Top 10 differentially expressed gene

Probe ID	Gene name	RUV-4			*t*-test		
		Log_2_ (fold change)	*p*-value^*^	*q*-value^*^	Log_2_ (fold change)	*p*-value	*q*-value
7927732	ARID5B	0.731982	2.30E-09	5.81E-05	0.426343	0.019245	0.232898
7925320	NID1	0.903102	1.02E-08	0.000129	0.365858	0.123152	0.347692
7926916	ZEB1	0.905861	1.40E-07	0.001182	0.358714	0.144518	0.368832
8145470	DPYSL2	0.696156	4.71E-07	0.002803	0.167631	0.3867	0.586169
8077528	SETD5	0.458547	5.54E-07	0.002803	0.502361	0.003411	0.226113
8143054	AKR1B1	0.720922	4.84E-06	0.020398	0.433627	0.053769	0.271417
7965123	PPP1R12A	0.654542	6.09E-06	0.020952	0.347578	0.103782	0.326709
8049487	MLPH	–0.88485	6.63E-06	0.020952	–0.00721	0.984862	0.992079
8119016	MAPK13	–0.54298	7.64E-06	0.021475	–0.07527	0.647691	0.783974
8141035	SGCE	0.654982	1.43E-05	0.036157	0.079062	0.722441	0.833266

^*^The variance of the statistic calculated by empirical variance.

Correlation between the expression of immunohistochemical markers and clinicopathological factors

The correlation between immunohistochemical patterns of E-cadherin and ZEB1 and clinicopathological factors was analyzed. Loss of E-cadherin (*p* = 0.015) and presence of ZEB1 expression in stromal cells (stromal ZEB1, *p* < 0.001) were significantly associated with degree of dysplasia (Table 3).

**Table 3:** Correlation between expression of immunohistochemical markers and clinicopathological factors

Parameters	E-cadherin			Epithelial ZEB1			Stromal ZEB1		
	No loss (*n* = 77)	Loss (*n* = 10)	*p*-value	Negative (*n* = 84)	Positive (*n* = 3)	*p*-value	Negative (*n* = 77)	Positive (*n* = 10)	*p*-value
Age (years, median ± SD)	63.6 ± 8.3	68.1 ± 7.7	0.103	64.0 ± 8.2	66.7 ± 11.2	0.585^†^	63.9 ± 8.6	65.3 ± 5.9	0.623
Sex (male)	47	6	0.949	51	2	0.836^*^	45	8	0.189
Dysplasia			0.015			0.107^*^			<0.001^*^
Noninvasive	64	5		68	1		67	2	
Invasive	13	5		16	2		10	8	
Subtype (noninvasive : invasive)									
Gastric	46:0	4:2	0.011^*^	49:1	0:2	0.002^*^	48:1	1:2	0.007^*^
Intestinal	9:4	1:2	0.518^*^	10:5	1:0	1.00^*^	10:4	0:2	0.125^*^
Pancreatobiliary	9:7	0:1	0.471^*^	9:8	0:0	NA	9:4	0:4	0.029^*^
Oncocytic	0:2	0:0	NA	0:2	0:0	NA	0:1	0:1	NA
T stage			0.268^*^			0.529^*^			0.316^*^
T1/T2	3	3		6	0		4	1	
T3/T4	10	2		10	2		6	7	
Lymph node metastasis	2	2	0.063^*^	3	1	0.133^*^	3	1	0.359^*^
Cyst size (≥30 mm)	37	3	0.331^*^	39	1	1.00^*^	36	4	1.00^*^
Diameter of pancreatic duct (mm, median ± SD)	5.3 ± 5.3	5.4 ± 4.9	0.957	5.3 ± 5.3	5.4 ± 3.9	0.976^†^	5.1 ± 5.3	6.8 ± 4.1	0.365

^*^Fisher’s exact test

^†^Mann-Whitney *U* test

SD, standard deviation; NA, not available.

Since DEGs were obtained by comparing low-grade dysplasia and invasive IPMN of the gastric subtype, subgroup analysis was performed according to histological subtypes. In the gastric subtype, loss of E-cadherin expression was observed more frequently in invasive IPMN (100.0%) as compared with noninvasive IPMN (8.0%, *p* = 0.011). ZEB1 expression in epithelial cells (epithelial ZEB1, 66. 7% vs. 0%, *p* = 0.002) and expression of stromal ZEB1 (66.7% vs. 2.0%, *p* = 0.007) were also significantly different between invasive and noninvasive IPMN. Stromal ZEB1 was more frequently expressed in invasive IPMN of the pancreatobiliary subtype (50.0% vs. 0%, *p* = 0.029). However, E-cadherin and epithelial ZEB1 did not show significant association with invasive IPMN in the subgroup analysis (Table 3). Both epithelial and stromal ZEB1 were expressed in two samples, and statistically relevant correlations were obtained between the epithelial and stromal ZEB1 expression (*p* = 0.002).

Positive staining of vimentin, though rarely seen, was found in 3.4% of the samples (*n =* 3). However, two of those three samples were stained less than 5% of the cytoplasm (grade 1) in non-neoplastic lesion of IPMN with intermediate-dysplasia. Only one sample of intermediate-dysplasia was stained as grade 2.

Logistic regression analysis revealed that loss of E-cadherin expression (*p* = 0.023, odd ratio [OR] = 4.923) and stromal ZEB1 (*p* < 0.001, OR = 26.800) were significantly correlated with degree of dysplasia. Epithelial ZEB1 expression showed marginal significance with degree of dysplasia (*p* = 0.088, OR = 8.500) (Table 4).

**Table 4:** Logistic regression analysis of immunohistochemical markers associated with increased risk of IPMN associated with an invasive carcinoma

	Odd ratio	95% CI	*p*-value
E-cadherin	4.923	1.244–19.482	0.023
Epithelial ZEB1	8.500	0.725–99.636	0.088
Stromal ZEB1	26.800	4.965–144.654	<0.001

CI, confidence interval

### Survival analysis

**Figure 5:**
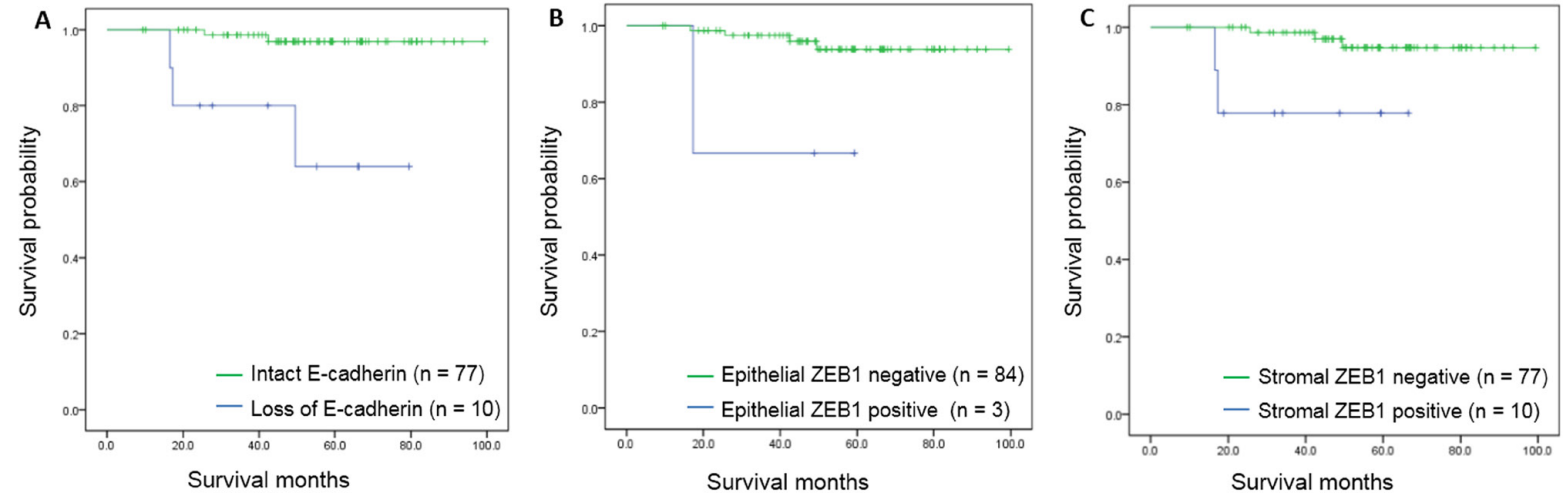
Kaplan-Meier survival curves for disease-specific survival according to immunohistochemical markers (**A**) Survival curves according to E-cadherin expression (*p* < 0.001). (**B**) Survival curves according to epithelial ZEB1 expression (*p* = 0.025). (**C**) Survival curves according to stromal ZEB1 expression (*p* = 0.009).

Disease-specific survival was significantly associated with expression of IHC markers. Poor disease-specific survival was found in patients with loss of E-cadherin expression (97.4 months vs. 62.2 months, *p =* 0.004) and expression of epithelial ZEB1 (96.5 months vs. 42.0 months, *p* < 0.001). Differences in survival rates were also correlated with expression of stromal ZEB1 (96.5 months vs. 56.6 months, *p* = 0.020) (Figure 5).

Using the Cox proportional hazards model, the hazard of death was found to be significantly increased in patients with loss of E-cadherin expression (hazard ratio [HR] = 13.718, *p* = 0.004), expression of epithelial ZEB1 (HR = 19.117, *p* = 0.001), and stromal ZEB1 (HR = 6.373, *p* = 0.043) (Table 5).

**Table 5:** Cox proportional hazard model analysis for disease-specific survival according to immunohistochemical markers

	Hazard ratio	95% CI	*p*-value
E-cadherin	13.718	2.280–82.519	0.004
Epithelial ZEB1	19.117	3.176–115.074	0.001
Stromal ZEB1	6.373	1.059–38.369	0.043

CI, confidence interval.

### Prognostic relevance of immunohistochemical markers

**Figure 6:**
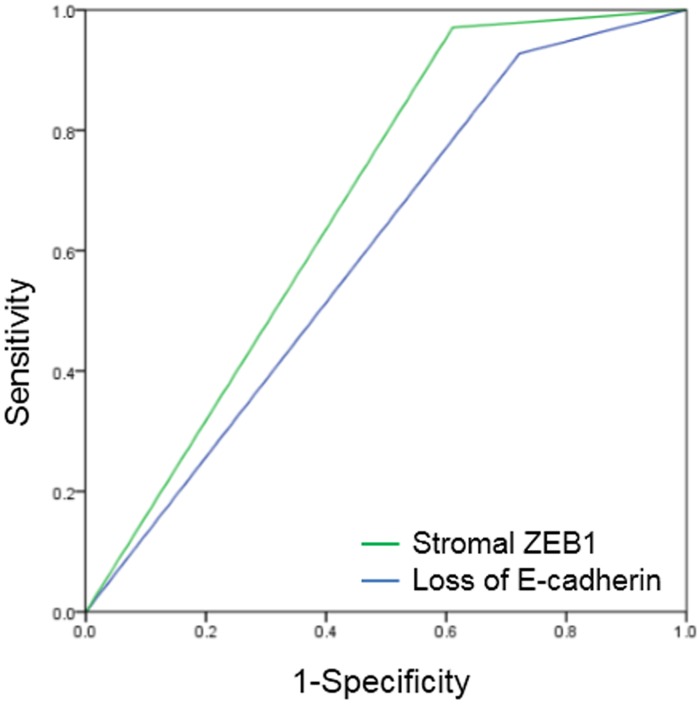
Receiver operating characteristic (ROC) curves of E-cadherin and stromal ZEB1 AUC for E-cadherin was 0.603 (*p* = 0.182), and stromal ZEB1 was 0.680 (*p* = 0.019).

In the receiver operating characteristics (ROC) curve analysis, the area under the curve (AUC) for E-cadherin and stromal ZEB1 was 0.603 (*p* = 0.182) and 0.680 (*p* = 0.019), respectively (Figure 6). Stromal ZEB1 demonstrated a sensitivity of 38.8% and a specificity of 97.1%. Positive prediction value (PPV) and negative prediction value (NPV) of stromal ZEB1 for prediction of poor survival was 77.8% and 85.9%, respectively.

## DISCUSSION

Epithelial-to-mesenchymal transition (EMT) is a multi-stage trans-differentiation process that epithelial cells undergo to attain the mesenchymal phenotype, which contributes to inflammation, wound healing, and carcinogenesis [11–13]. During EMT progression, epithelial cells lose their epithelial markers (such as E-cadherin, claudin, and laminin 1) and gain mesenchymal markers (such as vimentin and fibronectin) [14]. EMT is widely investigated in various cancers due to its association with tumor progression, metastasis, poor prognosis, and drug resistance [14–18].

One transcription factor that orchestrates the EMT is ZEB1, which is encoded by the TCF8 gene, and is the vertebrate homologue of the ZFH gene family of zinc finger/homeodomain proteins. It is known to be a key mediator of EMT, and induces EMT by inhibiting expression of E-cadherin and miRNAs [19–23]. Previous studies have demonstrated that expression of ZEB1 is correlated with advanced tumor grade and poor outcomes in pancreatic cancer [24–29]. However, little is known about the role of EMT in IPMN. Gene expression changes associated with EMT during the progression of IPMN was first examined in a study by gene set enrichment analysis (GSEA) and Ariadne sub-network analysis in 28 IPMN samples [10]. Lahat *et al.* demonstrated that positive EMT status was associated with reduced disease-free survival in IPMN patients, suggesting that the EMT markers could be utilized as potential biomarker in IPMN [11]. Expression of Twist and Bmi1, which are involved in the initiation and execution of EMT, was also reported to be associated with aggressiveness and poor prognosis of IPMN [30]. In accordance with these data, loss of E-cadherin expression (OR = 4.923, *p* = 0.023) and expression of stromal ZEB1 (OR = 26.800, *p* < 0.001) was correlated with degree of dysplasia. Disease-specific survival differed significantly according to different according to the loss of E-cadherin expression (97.4 months vs. 62.2 months), expression of epithelial ZEB1 (96.5 months vs. 42.0 months), and stromal ZEB1 (96.3 months vs. 56.6 months). These results suggested that E-cadherin and ZEB1 may be used as prognosticators of IPMN.

The incidence of positively stained vimentin in the present study was lower than of 6.0% [29] and 58.0% [11] incidence observed in previous studies. EMT involves progressive loss of epithelial markers and gain of mesenchymal markers during the functional transition of polarized epithelial cells into mobile and ECM component-secreting mesenchymal cells [14]. According to previous study, mesenchymal markers such as vimentin was overexpressed in the poorly differentiated and not in the well-differentiated pancreatic carcinomas among the overall 11.8% of positive staining of vimentin [31]. Since IPMN with various degrees of dysplasia were included in this study, vimentin expression was scarce and did not qualify as a prognostic predictor for IPMN in the present study.

In this study, ZEB1 expression in the epithelial cell was observed only in 3 cases (3.4%), which is comparable with that reported in a previous study (2%) [29] and consistent with the notion that cancer stem cells occur rarely in the tumor cell population. In regards to stromal ZEB1, ZEB1 has been previously reported to be found in the stromal fibroblasts surrounding the epithelial tumor cells [32, 33]. The functional contributions of stromal cells have been actively investigated since tumor growth is thought to be dependent on the dynamic interaction with adjacent stromal cells that might in turn compromise the tumor microenvironment [34]. There is increasing evidence that stromal cells play a crucial role in the stimulation of epithelial cancer cell growth, paracrine induction of EMT, invasion, and metastasis by interacting with tumor cells [35–38]. In breast carcinomas, expression of ZEB1 has been reported to occur in the stromal compartment of supposing to represent two populations of cells: EMT-transformed neoplastic cells and stromal fibroblastic cells undergoing activation of ZEB1 [39]. Expression of transcription factor ZEB1 in the stromal cells has been proven to be associated with EMT and tumor progression in urothelial [40] and endometrial cancer [33]. Previously, there have been two studies that investigated the prognostic value of stromal ZEB1 in pancreatic cancer [29, 41]. In accordance with those reports, stromal ZEB1 showed correlation with epithelial ZEB1, and was associated with significantly increased risk of invasive IPMN and poor survival in the present study. Stromal ZEB1 had the greatest AUC value of 0.680, with PPV of 77.8% and NPV of 85.9% which implicated that stromal ZEB1 could be a strong prognostic factor of IPMN. To the best of our knowledge, this study is the first attempt to evaluate and validate the clinical relevance of stromal ZEB1 expression in IPMN, which was identified in gene expression profiling.

Several studies have suggested that different clinical characteristics and distinct pathways to malignant progression exist among the various IPMN subtypes. Genetic alteration such as mutational status of *GNAS* or *KRAS* [42–44], as well as expression pattern of mucin [42, 43, 45, 46] have been investigated. Recent meta-analysis suggested that subtype identification should be considered in future guidelines for management of IPMN, since subtypes have an impact on disease prognosis [47]. On the contrary, some investigators have shown that histological subtypes have limited prognostic value for pancreatic IPMN, although histological subtypes are associated with degree of dysplasia [48]. This is a controversial topic, and is the subject of much debate. Moreover, several pitfalls exist in the molecular study of IPMN; a single IPMN lesion sample may contain multiple subtypes with varying degrees of dysplasia, from low-grade to invasive carcinoma. Furthermore, the epithelium suitable in quantity and quality for diagnosis and further investigational studies may only reflect a small part of the lesion.

Therefore, several efforts were made in this study to overcome the present challenges. Since gene expression of housekeeping genes remained poorly normalized following RMA normalization, the RUV method was used in this study. The RUV normalization method was developed to reduce errors in gene expression profiling due to unknown structural errors [49]. To reduce unwanted variation, the RUV method decomposes expression patterns of known control genes (e.g. housekeeping genes) into several factors via factor analysis, and normalizes the expression of genes of interest using these factors as covariates. As a result, it yields more accurate estimates of expression fold-changes as compared with other normalization methods [50]. Among the various normalization methodologies, the RUV-4 method was used in this study, which is known to give more stable results [51]. Empirical variance adjustment was performed to calculate the *p*-values, and to identify DEGs. In addition, low-grade and invasive IPMN of the gastric subtype were compared for identification of DEGs. This was done to exclude biases resulting from possible subtype-associated heterogeneity; subgroup analysis of immunohistochemical results was also performed. However, owing to the limited number of samples and the inherently low epithelial ZEB1 expression levels, subgroup analysis failed to show statistical significance in intestinal and pancreatobiliary subgroups for several assessed factors.

This study has several limitations. The present study is the first to report the prognostic relevance of ZEB1 expression in IPMN, and supports the hypothesis that treatment strategies targeting ZEB1 may also target tumor cells, as well as stromal cells. However, no further information can be inferred regarding the mechanism from this study. Further investigations into the underlying mechanisms may contribute to better management of IPMN. Gene expression profiling was limited to the gastric subtype to reduce heterogeneity. However, this study does not provide information regarding the genetic changes associated with subtypes in the malignant progression of IPMN.

For clinical application of this marker, preoperative use may be limited due to the low sensitivity of current diagnostic modalities. A recent study demonstrated that the overall diagnostic accuracy of endoscopic ultrasound-guided fine needle aspiration ranges from 54% to 97%, and may be lower in smaller cysts [52]. Malignancy within a cystic neoplasm can be identified with 25% to 88% sensitivity [52]. Endoscopic retrograde cholangio-pancreatography tissue sampling also has a relatively low diagnostic yield; a pooled sensitivity of 35.1% [53]. However, molecular analysis in combination with imaging and clinical features was better able to characterize the malignant potential of pancreatic cysts compared to either test alone [54].

In conclusion, gene expression profiling was performed to determine DEGs associated with malignant progression of IPMN and the association of EMT with the prognosis of IPMN was evaluated. Particularly, the clinical relevance of stromal ZEB1 expression in IPMN was investigated inceptively. Loss of E-cadherin and expression of stromal ZEB1 were associated with increased risk of invasive IPMN. Stromal ZEB1, as well as epithelial ZEB1, and E-cadherin were strong predictors of survival in patients with IPMN. Our findings suggest that these EMT markers may be utilized as potential prognosticators and may be used to improve and personalize treatment of IPMN.

## MATERIALS AND METHODS

### Patient and tissue samples

This study was approved by the Institutional Review Board of the Seoul National University Hospital (H-1309-024-517), and written informed consents were obtained from all participants prior to study initiation. A total of 76 fresh-frozen IPMN tissues were collected from patients who underwent pancreatic resection at the Seoul National University Hospital between June 2009 and February 2013. Tissues were selected for the present study based on the quantities of neoplastic cells. For validation of DEGs, formalin-fixed, paraffin-embedded blocks from 87 patients diagnosed with IPMN following pancreatic resection at the Seoul National University Hospital between April 2002 and October 2009 were selected. The IPMNs were classified into 4 histopathological subtypes; gastric, intestinal, pancreatobiliary, and oncocytic [55]. Degree of dysplasia was classified according to the guidelines set by the World Health Organization classification of Tumors of the Digestive System [56], and were categorized as low-grade dysplasia, intermediate-grade dysplasia, high-grade dysplasia, and IPMN with an associated invasive carcinoma. Staging was examined according to the 7th edition of the American Joint Committee on Cancer staging manual [57]. Clinicopathological data including demographics, radiologic imaging, and histopathological data were prospectively collected in electronic medical record forms. Tissue slides were thoroughly reviewed by a specialized gastrointestinal pathologist (K.B.L.).

### mRNA microarray

In the present study, global gene expression was analyzed using the Affymetrix GeneChip^®^ Human Gene 1.0 ST oligonucleotide arrays (Affymetrix). Following the operation, a 1 mm^3^ sized tumor tissue was immediately collected and stored in a −70°C liquid nitrogen tank until RNA extraction. Total RNA was isolated using the RNeasy Mini Kit columns (Qiagen, Hilden, Germany) according to manufacturer’s instructions. Total RNA (300 ng) from each sample was converted to double-stranded cDNA using a random hexamer incorporating a T7 promoter, and amplified RNA (cRNA) was generated from the double-stranded cDNA template though an *in vitro* transcription reaction; RNA samples were purified with the Affymetrix sample cleanup module. cDNA was generated through random-primed reverse transcription using a deoxynucleotide-triphosphate (dNTP) mix containing deoxyuridine-triphosphate (dUTP). The cDNA was then fragmented by uracil-DNA glycosylase (UDG) and apurinic/apyrimidinic (AP) 1 restriction endonucleases, and end-labeled by terminal transferase reaction with biotinylated dideoxynucleotide. Fragmented end-labeled cDNA was hybridized to the GeneChip® Human Gene 1.0 ST arrays for 16 hours at 45°C and 60 rpm, as described in the Gene Chip Whole Transcript (WT) Sense Target Labeling Assay Manual (Affymetrix). After the final wash and staining step, Affymetrix GeneChip^®^ Human Gene 1.0 ST oligonucleotide array was scanned using the Affymetrix Model 3000 G7 scanner. Image data was extracted using the Affymetrix Commnad Console software 1.1 (Affymetrix). Expression data were generated by the Affymetrix Expression Console software version1.1 (Affymetrix). RNA concentration was calculated via a spectrophotometer; the purity and integrity of isolated RNA were evaluated by OD260/280 for quality control. In total, 76 samples passed the quality control test, and were selected for further analysis.

### Identification of differentially expressed genes

RUV-4 and empirical variance adjustments were performed to determine the DEGs. In order to carry out the RUV method, 2 components were determined. The first component was the selection of control genes in the RUV. Rubie *et al.* [58] experimentally demonstrated that expression patterns of genes known as housekeeping genes in pancreatic cancer tissues were often different from those in normal tissues. Therefore, 10 control genes were specifically selected based on results of Rubie *et al.* [58] (18S rRNA, QRRS, PMM1, POLR2L, GUS, TAF2, SDHA, PSMB6, ADA, and UBE2D2); these genes were shown to have comparable expression patterns between pancreatic cancer and normal tissues. The RLE plots of control genes with 1–8 factors were compared to determine the appropriate number of factors that was consistent with the gene expression patterns of the control genes. The differences in gene expression between low-grade dysplasia and invasive IPMN in the control genes were also assessed, and the minimal number of factors required to yield a *p*-value of > 0.2 for all control genes was determined. After selecting the number of factors that satisfied the above two conditions, RLE boxplots for all genes were obtained, and the variance of the statistic was calculated. All analyses were carried out using the R program ver. 3.3.1 and package “ruv”.

### Immunohistochemistry

Immunohistochemical staining was performed at the SuperBioChips Laboratories, Seoul, Korea, to validate the differential expression of selected genes. In order to exclude bias due to possible tumor heterogeneity, 2 punches from the invasive tumor or highest grade dysplasia and 1-2 punches from lower grade dysplasia or non-neoplastic pancreas were obtained from each patient.

Tissue sections (4-μm thick) were cut from each formalin-fixed, paraffin-embedded block, and were deparaffinized and rehydrated. Heat-induced antigen retrieval was performed at 100°C for 24 minutes in cell conditioning 1 solution (Ventana Medical Systems, AZ, USA). Slides were incubated in 3% hydrogen peroxidase for 4 minutes. Immunohistochemical staining was performed using the Optiview DAB IHC Detection Kit (Ventana Medical Systems, AZ, USA). The primary antibodies used were as follows: E-cadherin (BD Biosciences, San Jose, CA, USA, mouse monoclonal, 1:800) and ZEB1 (Novus biologicals, Littleton, CO, USA, rabbit polyclonal, 1:100). Reactions were detected with a diaminobenzidinetetrahydrochloride solution (Biogenex, San Ramon, CA, USA), and hematoxylin counterstaining was performed for 8 minutes.

### Scoring of immunohistochemistry

**Figure 7:**
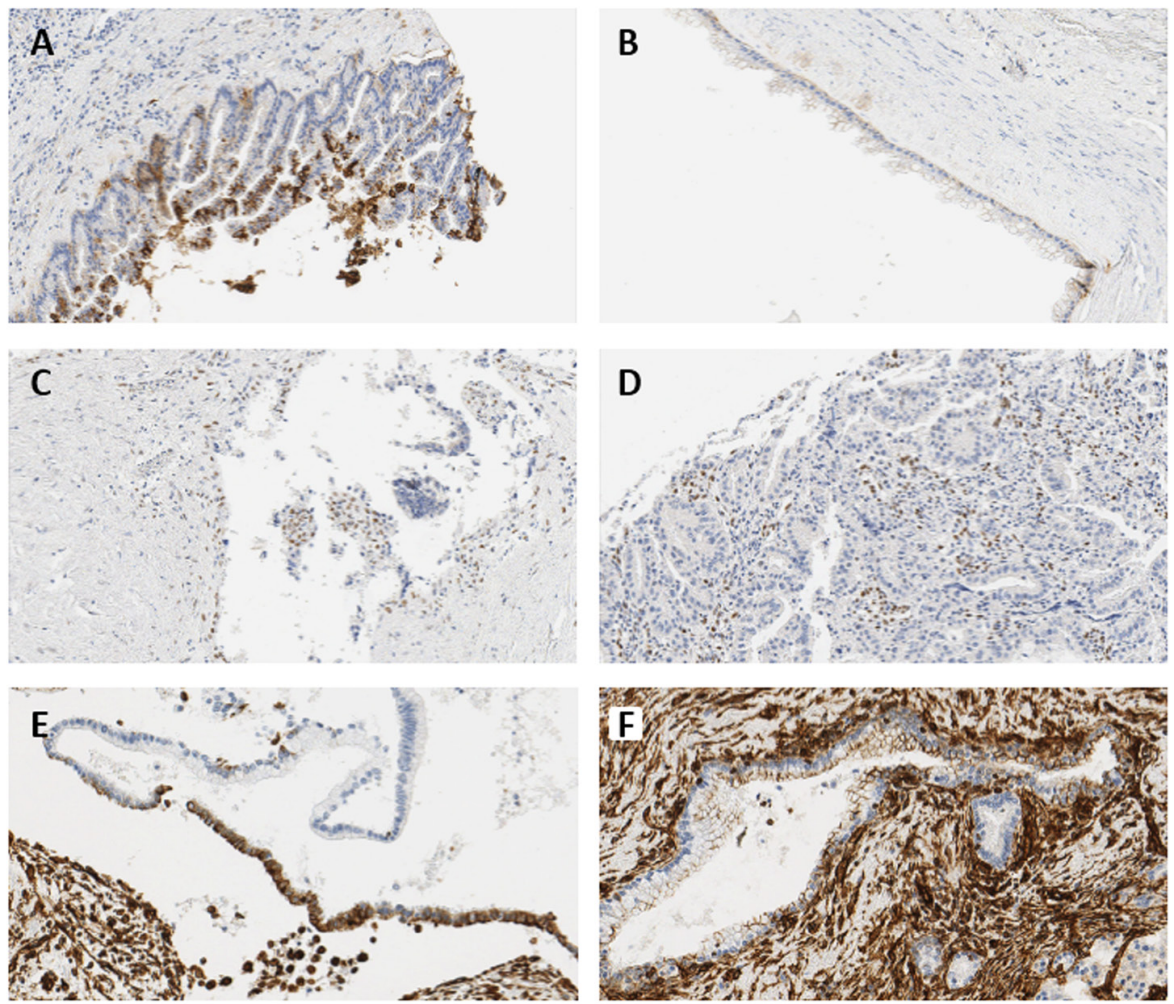
Representative immunohistochemical images showing expressions of E-cadherin and ZEB1 (**A**) 5–50% loss of E-cadherin in IPMN with high-grade dysplasia, 224× magnification (grade 1). (**B**) ≥50% loss of E-cadherin in IPMN with moderate-grade dysplasia, 232× magnification (grade 0). (**C**) Nuclear expression of ZEB1 in the epithelial cells of invasive IPMN, 264× magnification (intensity grade 2, area grade 3, score 6). (**D**) Positive stromal ZEB1 expression in invasive IPMN, 264× magnification. (**E**) 5–50% expression of vimentin in IPMN with moderate-grade dysplasia, 264× magnification (grade 2). (**F**) <5% expression of vimentin in non-neoplastic lesion of IPMN with moderate-grade dysplasia, 264× magnification (grade 1).

Since there is no generally accepted classification for immunohistochemical ZEB1 expression, authors defined the grades of immunohistochemical staining as follows: E-cadherin expression was evaluated according to the percentage of cells that were labeled, and was categorized as negative when expression was normal (grade 0), and positive when grade was 1–3. The intensity of ZEB1 expression in the epithelium was graded as follows; 0: completely negative; 1: rare dots observed under high magnification; 2: easily recognized dots under high magnification; 3: easily recognized dots under moderate magnification; 4: easily recognized dots under low magnification [41]. Scores of ZEB1 expression in the epithelium were determined by multiplying the grade of the dominant staining intensity by the grade of the area; values were categorized as negative when score was 0 or 1, and positive when score was greater or equal to 2. Representative figures are shown in Figure 7. ZEB1 expression in tumor stroma was classified as positive or negative. Vimentin expression was evaluated with respect to the percentage of cells labeled, and was categorized under grades 0–3 (Table 6).

**Table 6:** Scoring criteria of E-cadherin, ZEB1, and vimentin

Grade	E-cadherin	ZEB1			Vimentin
		Intensity	Area	Stromal cell	
**0**	normal	Negative	Negative	Negative	Negative
**1**	<5% loss	Equivocal	<5%	Positive	<5%
**2**	5–50% loss	Weak	5–50%		5–50%
**3**	≥50% loss	Moderate	≥50%		≥ 50%
**4**		Strong			

### Statistical analysis

Categorical variables were compared via the Chi-squared or the Fisher’s exact test to examine associations between immunohistochemical expression and clinicopathological factors. For comparison of continuous variables, Student’s *t*-test or the Mann-Whitney *U* test was used. Logistic regression analysis and Cox proportional hazards model were also performed. For evaluation of predictive parameter values, positive and negative predictive values were calculated from cross-tables and ROC curves; AUCs were calculated. Difference in survival time was calculated by the Kaplan-Meier method, and the log-rank test was applied. *P*-values of less than 0.05 were considered to be statistically significant. The IBM SPSS Statistics version 20 (SPSS Inc, Chicago, IL) software was used for analyses.

## Abbreviations

AUC: area under the curve
DEG: differentially expressed gene
EMT: epithelial-to-mesenchymal transition
HR: hazard ratio
IPMN: intraductal papillary mucinous neoplasm
IPMN with and invasive carcinoma: invasive IPMN
IQR: inter quartile range
NPV: negative prediction value
OR: odds ratio
PPV: positive prediction value
RLE: Relative Log Expression
RMA: Robust Multi-Array Average
ROC: receiver operating characteristics
RUV: removing unwanted variation
ZEB1: zinc finger E-box binding homeobox 1
